# Production and *in vivo* PET/CT imaging of the theranostic pair ^132/135^La

**DOI:** 10.1038/s41598-019-47137-0

**Published:** 2019-07-23

**Authors:** Eduardo Aluicio-Sarduy, Reinier Hernandez, Aeli P. Olson, Todd E. Barnhart, Weibo Cai, Paul A. Ellison, Jonathan W. Engle

**Affiliations:** 1 Department of Medical Physics, University of Wisconsin-Madison, Madison, WI 53705 USA; 2 Department of Radiology, University of Wisconsin-Madison, Madison, WI 53705 USA

**Keywords:** Nuclear chemistry, Cancer imaging

## Abstract

The present study describes a novel method for the low energy cyclotron production and radiochemical isolation of no-carrier-added ^132/135^La^3+^ from bulk ^nat^Ba. This separation strategy combines precipitation and single-column extraction chromatography to afford an overall radiochemical yield (92 ± 2%) and apparent molar activity (22 ± 4 Mbq/nmol) suitable for the radiolabeling of DOTA-conjugated vectors. The produced ^132/135^La^3+^ has a radiochemical and radionuclidic purity amenable for ^132^La/^135^La-based cancer theranostic applications. Longitudinal PET/CT images acquired using the positron-emitting ^132^La and *ex vivo* biodistribution data separately corroborated the accumulation of unchelated ^132/135^La^3+^ ions in bone and the liver.

## Introduction

**Figure 1:**
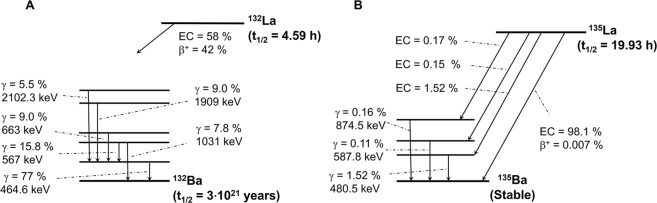
Simplified decay schemes of ^132^La (**A**) and ^135^La (**B**)^4,15,16^.

Targeted Radionuclide Therapy (TRT) using electron-emitting radiometals has shown efficacy in the treatment of several malignancies. However, due to the long range of β- emissions, treatments often cause significant toxicities to normal surrounding tissues. Conversely, given the high linear energy transfer (LET) of Auger electrons, isotopes decaying by electron capture have the potential to locally deposit dose in target tissue while sparing normal tissues. In this regard, ^135^La (t_1/2_ = 19.93 h, 100% EC) is promising due to its suitable decay characteristics (Fig. 1A) and chemical properties resembling other common therapeutic radiometals (e.g., ^177^Lu, ^90^Y or ^225^Ac). Low energy proton irradiation of natural barium generates a mixture of ^132-136^La (^13x^La) radionuclides including ^135^La and the positron-emitting ^132^La (t_1/2_ = 4.59 h) (Fig. 1B) via ^nat^Ba(p,x)^13x^La reactions. Reported production cross-sections for ^13x^La radioisotopes from 12–70 MeV of ^nat^Ba targets show that differential decay kinetics form ^135^La with high radionuclidic purity at energies available on most medical cyclotrons^1–4^. Furthermore, small quantities of co-produced, positron-emitting ^132^La enable seamless implementation of a theranostic approach and the noninvasive interrogation of pharmacokinetic profiles by *in vivo* positron emission tomography imaging and *ex vivo* biodistribution. To date, relatively few separation strategies have been reported for the radiochemical isolation of no-carrier-added lanthanum from barium, and they have achieved only moderate separation factors and chemical purity^3–5^. Our goal was to develop an optimized production method for ^132/135^La with a chemical purity suitable for chelation and *in vivo* PET imaging of radiolabeled, targeted theranostic pharmaceuticals.

## Materials and Methods

Barium target material was purchased from Sigma-Aldrich with a purity of 99.9% (trace metal basis) and stored in inert gas atmosphere. Optima grade HNO_3_ and HCl from Fisher Chemical were used for the radiochemical separation studies. All glassware was washed with Alconox® solution, rinsed with 1 M HNO_3,_ water (18 M Ohm cm^−1^), and finally dried at 120 °C to ensure the removal of trace metals.

### Target preparation and irradiation

^13x^La were co-produced by proton irradiation of ^nat^Ba targets (~450 mg) via ^nat^Ba(p,n)^13x^La reactions using a 16 MeV GE PETtrace cyclotron. To make the targets, metallic barium was pressed (50 kg cm^−2^) into a niobium crucible with 12.2 mm diameter, 1.2 mm deep pocket. As target preparation was performed in air, the target was immediately installed on the cyclotron and exposed to high vacuum to limit barium oxidation. For imaging studies, irradiations were performed at 10 µA for up to 3 h with direct water cooling to the back of the niobium target holder, and a 0.25 mm Nb foil was used to degrade the incident beam energy from 16 MeV to 11.9 MeV. Irradiations were also performed at nominal 16 MeV beam energy and a maximum current of 25 µA for up to 1.5 h to compare production yields and to investigate additional (p,2n)-type nuclear reactions’ effect on radioisotopic purity.

### Radiochemical isolation of ^132/135^La

The separation of ^132/135^La from the irradiated ^nat^Ba targets was carried out by combining a precipitation method with column extraction chromatography using an N,N,N’,N’-tetrakis-2-ethylhexyldiglycolamide functionalized resin (DGA-branched, Eichrom). Between irradiation and the start of the separation, a 2–3 h “cool down” period decayed co-produced ^134^La (t_1/2_ = 6.5 min) and ^136^La (t_1/2_ = 9.9 min). Afterward, 5 mL of 6 M HNO_3_ dissolved ^132/135^La while precipitating bulk Ba as Ba(NO_3_)_2_. Following centrifugation, the supernatant was passed through a 1 ml fritted solid phase extraction (SPE) tube filled with ~130 mg of branched DGA resin, trapping ^132/135^La^3+^ and eluting Ba^2+^. Washing with decreasing concentrations of HNO_3_ (3–0.5 M) eluted trace metal impurities of Cu, Fe and Zn. Finally, no-carrier-added ^132/135^La^3+^ was eluted in a small volume of dilute 0.1 M HCl (4 × 300 µL). The loading, rinsing, and elution steps were carried out using a peristaltic pump at a flow rate of 1.6 mL min^−1^. The activity was quantified using an efficiency calibrated high purity germanium (HPGe) detector (10%, Al-window, 1.9 keV FWHM at 1333 keV).

### Radiochemical and chemical analysis

Radiochemical yields were followed by HPGe gamma-ray spectrometry. Trace metal content of the chromatography fractions was measured with an Agilent 4200 microwave plasma-atomic emission spectrometer (MP-AES). Calibration curves were generated using commercially available multielement standards (Sigma-Aldrich). For transition metals, the typical detection limits of this technique are in the ppb range.

### Apparent molar activity quantification

The apparent molar activity, an indication of the chemical purity of the produced lanthanum, was measured as described previously^6,7^. Briefly, the ability of DOTA to complex ^13x^La^3+^ ions was determined by incubating aliquots of ^135^La^3+^ (3.7 MBq) with increasing DOTA concentrations (0–100 µg/mL) in 0.5 M NaOAc buffer solution (pH = 4.5) for 30 min at 80 °C. The complexation yield for each ^135^La^3+^/DOTA ratio was determined by autoradiographic thin layer chromatography (radio-TLC) using silica-impregnated paper as stationary phase and 1:1 MeOH:10% NH_4_OAc (w/v) as the mobile phase. Radioactivity distribution was visualized on the TLC plates using a Packard Cyclone phosphor plate reader. To compute the apparent molar activity, ^135^La^3+^ activity in MBq was divided by twice the number of moles of DOTA required to complex 50% of the radioactivity, and the value was reported in MBq/nmol (mean ± standard deviation, SD).

### Positron-emission tomography (PET) imaging and *ex vivo* biodistribution

For *in vivo* distribution studies, “free” ^132/135^La^3+^ was prepared for injection by buffering the activity in phosphate buffered saline (PBS) containing 0.05 M sodium acetate. The weakly chelating acetate ion was added to avoid the formation of La radiocolloid at physiological pH (Fig. S1, Supporting Information). Animal experiments were conducted with the approval of the University of Wisconsin Institutional Animal Care and Use Committee (IACUC). All studies were conducted in accordance with the relevant guidelines and regulations. To assess the *in vivo* biodistribution of “free” La^3+^ ions, positron emission tomography (PET) imaging was performed in 10-week-old female ICR mice injected intravenously with 0.93 MBq of “free” ^132^La^3+^. Longitudinal static PET scans (photon energy window = 350–650 keV; coincidence timing window = 3.432 ns; axial resolution at center of FOV = 1.5 mm) collecting 40 million counts each were acquired of the anesthetized mice (2% isoflurane) at 0.5, 2, 5, and 20 h post-injection (p.i.) using an Inveon micro-PET/micro-CT scanner (Siemens). CT images were employed for anatomical co-registration and attenuation correction (80 kV, 900 μA, resolution 105 μm). Quantification of decay corrected PET/CT images was performed in an Inveon Research Workstation by manually drawing volume-of-interest (VOI) over the heart, muscle, bone, liver, and kidney. Quantitative data was expressed as percent injected activity per gram of tissue (%IA g^−1^; mean ± SD).

Following the last PET scan, *ex vivo* tissue distribution studies were performed for comparison with PET results. Mice were sacrificed, and organs were collected, wet-weighed, and counted in a calibrated gamma counter using an energy window from 10 to 100 keV (Wizard 2, PerkinElmer). Tissue radioactivity concentrations were calculated and reported as percent injected activity per gram of tissue (%IA g^−1^; mean ± SD).

## Results and Discussion

### Irradiation and production yield

The irradiation of the target was performed at a proton energy of 11.9 MeV and a beam current of 10 µA for 1.5–3 h. Under these irradiation conditions, ^135^La was produced with an end-of-bombardment (EOB) physical yield of 5.6 ± 1.1 MBq μAh^−1^ (n = 3). Due to the natural isotopic composition of the target, short-lived ^134^La and ^136^La were co-produced but at the end of chemistry (EOC) (ca. 5 h post-EOB), had decayed below the detection limit^3^. Relatively longer-lived ^132^La was also co-produced with a yield of 0.26 ± 0.05 MBq μAh^−1^ (~5% relative to ^135^La activity at EOB). Irradiations carried out at 16 MeV resulted in a marked increase in ^135^La production yields (16.4 ± 1.1 MBq μAh^−1^) but resulted in a reduction in the radionuclidic purity due to the co-production of the positron-emitter ^133^La (t_1/2_ = 3.91 h). Production yields of ^132/133/135^La at the two different proton irradiation energies are summarized in Table S1 (Supporting Information).

### Separation of ^13x^La from ^nat^Ba targets

Following irradiation and target “cool down”, 5 mL of 6 M HNO_3_ was added to dissolve the produced ^132/135^La while precipitating bulk Ba as Ba(NO_3_)_2 (s)_. Ba(NO_3_)_2_ is a soluble salt, but in the presence of concentrated HNO_3_ the solubility significantly decreases with increasing acid concentration^8^. In 6 M HNO_3_ the solubility of Ba(NO_3_)_2_ is about 2.4 mg ml^−1^, allowing the precipitation of the bulk Ba (~99%) target material. After centrifugation, the supernatant was passed through a branched DGA resin which retained ^132/135^La^3+^ but not the remaining Ba^2+^ ions. Two subsequent rinses of the precipitate with 5 mL of 3 M HNO_3_ were loaded onto the column to ensure the maximum recovery of lanthanum.

The loaded column was then rinsed with decreasing concentrations of HNO_3_ (3–0.5 M), eluting the trace metal impurities of Cu, Zn and Fe. Efficiently removing these metal contaminants is essential to obtain elevated radiolabeling yields, especially when non-specific chelators such as DOTA are employed. The quantitative removal of metal impurities was possible given their low affinity constant (K_d_ < 2) over a wide range of HNO_3_ concentrations for the branched DGA resin^9,10^. A final 0.5 M HNO_3_ rinse was performed to decrease the acidity of the column bed while preventing ^132/135^La^3+^elution from the column.

**Figure 2:**
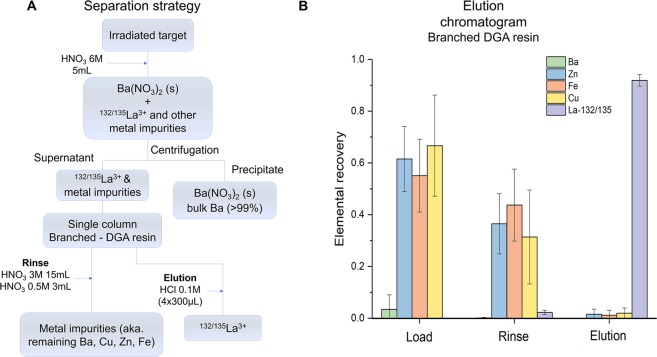
(**A**) Simplified separation strategy, (**B**) Elution profile from the branched - DGA resin as measured by MP-AES (Ba, Zn, Fe, and Cu) and HPGe (^132/135^La).

Due to the low affinity of the DGA resin for La^3+^ in 0.1 M HCl, ^132/135^La^3+^ was eluted in a small volume (<600 µL), with a final recovery efficiency of 92 ± 2% (n = 6) and a Ba/La separation factor of 10^6^. This separation factor is four orders of magnitude higher than those reported in previous separation strategies^3^. Figure 2 summarizes the separation strategy and the elution profile from the branched DGA column.

### Radionuclidic and chemical impurities

**Figure 3:**
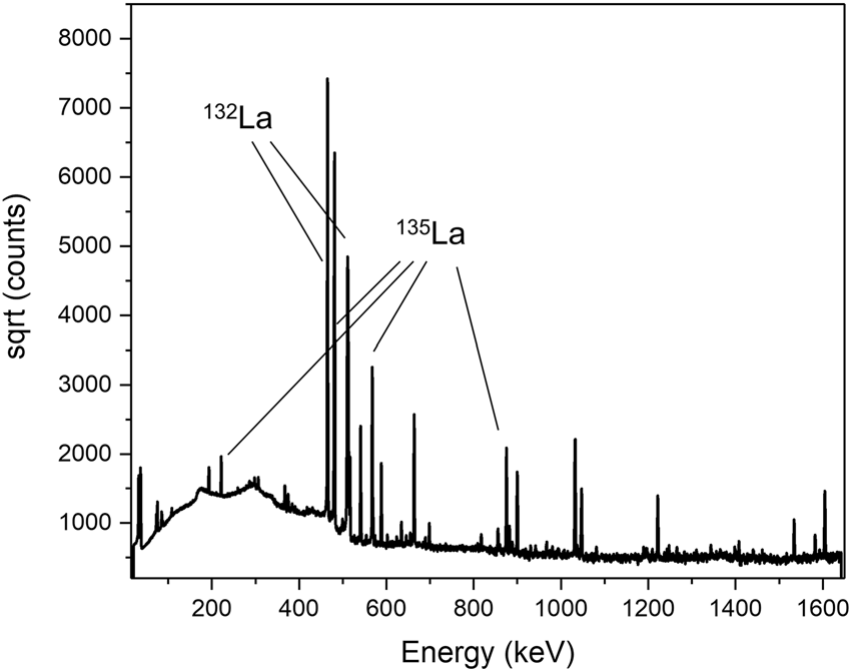
W Gamma spectrum at EOC of the ^132/135^La final eluate. All unlabeled low-intensity peaks correspond to additional ^132^La emissions.

The isotopic composition of the natural Barium target (0.11% ^130^Ba; 0.10% ^132^Ba; 2.42% ^134^Ba; 6.59% ^135^Ba; 7.85% ^136^Ba; 11.23% ^137^Ba; and 71.70% ^138^Ba) bears weight on the radionuclidic composition of the final isolate. As measured by γ-ray spectrometry (Fig. 3) at EOC (ca. 5 h post-EOB), besides ^135^La, the other detectable lanthanum isotope was the positron-emitting ^132^La (2.47%). As discussed in section 3.5, the co-produced positron-emitting ^132^La was leveraged to describe the biodistribution of the therapeutic ^135^La^3+^ *in vivo*. In future therapeutic applications requiring higher ^135^La production yields, the irradiations can be performed at higher energies (i.e. 16 MeV) using enriched target material which will avoid the co-production of radionuclidic impurities. Nevertheless, for initial preclinical evaluations, our ^135^La production method from ^nat^Ba and its associated radionuclidic purity is entirely feasible.

MP-AES determination of Ba and other transition metals influencing the ^132/135^La^3+^ apparent molar activity, including Cu, Zn and Fe was 638 ± 148 ppb, 64 ± 40 ppb, 138 ± 97 ppb, and <50 ppb, respectively (n = 3). The efficient removal of the target material and other metallic impurities (e.g., Cu, Zn, and Fe) played a decisive role in the obtained radiolabeling yields, as carboxylate chelating agents typically show high thermodynamic stability constants with these ubiquitous transition metals. The ppb levels of all these metals found in the final eluate did not preclude a high ^132/135^La^3+^ apparent molar activity with common chelators such as DOTA.

### Apparent molar activity using the DOTA chelator

The use of chelating agents like DOTA is a common method to probe the reactivity of produced radiometals^6,7,11^. The non-specific character of DOTA allows for the effective formation of stable complexes with many metal ions^12^, providing practical information about the chemical purity of the produced radiometal. The ^135^La^3+^ apparent molar activity obtained by the titration of ^135^LaCl_3_ solutions with DOTA was 22 ± 4 MBq (^135^La) nmol^−1^ (n = 3), sufficient for labeling biological targeting vectors with ^132/135^La^3+^ for potential theranostic applications.

### *In vivo* and *ex vivo* biodistribution

**Figure 4:**
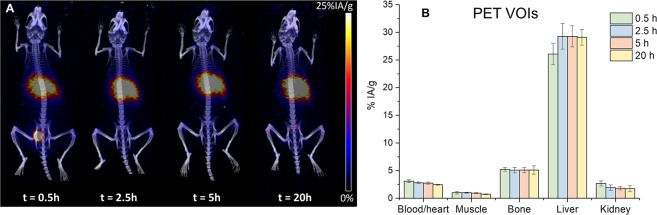
(**A**) Maximum intensity projection (MIP) static PET images of a representative ICR mouse injected intravenously with ^132/135^La^3+^. (**B**) ^132/135^La tissue uptake quantification of hand-drawn PET VOIs in ICR mice (n = 3, mean ± SD) injected with a rapid intravenous bolus of ^132/133^La^3+^.

For the first time, the evaluation of the *in vivo* biodistribution of ^132/135^La^3+^ was performed in normal ICR mice (n = 3) by following the ^132^La positron emissions via longitudinal PET/CT. Figure 4A shows representative coronal maximum intensity projection (MIP) PET images at 0.5, 2, 5 and 20 h after intravenous injection of the unchelated ^132/135^La^3+^. Quantitative VOI analysis was performed to quantify the radionuclide accumulation values in all major organs/tissues. In all four time points, liver and bone showed persistent ^132/135^La^3+^ uptake, higher than that seen in other normal tissue including heart/blood, kidneys, and muscle. As seen in Fig. 4B (Table S2, Supporting Information), liver uptake peaked at 29.28 ± 2.32 %IA g^−1^ at 2.5 h post-injection and bone showed highest activity accumulation (5.23 ± 0.33 %IA g^−1^) at the earliest measured timepoint 0.5 h after injection.

**Figure 5:**
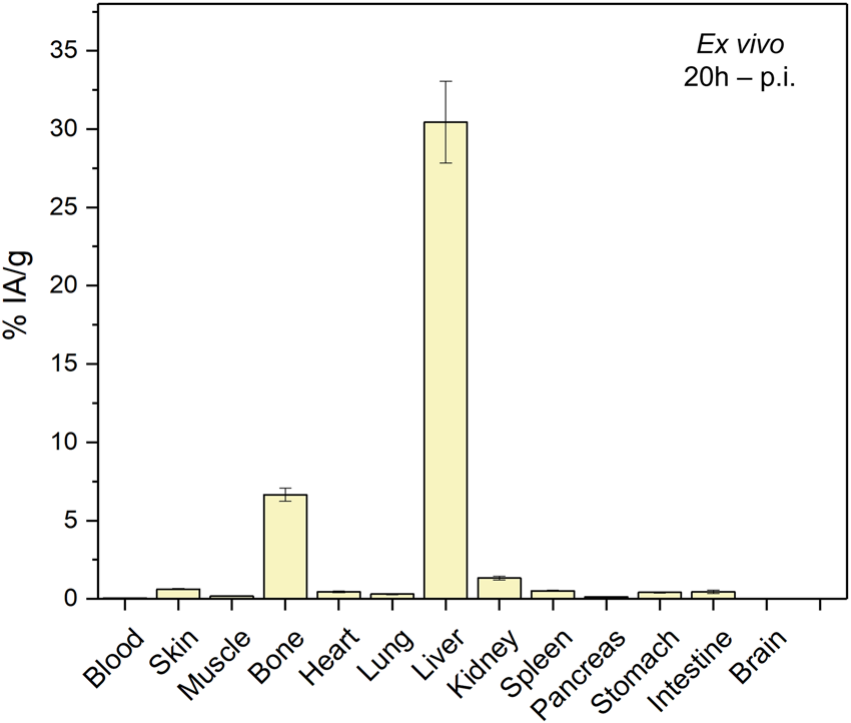
*Ex vivo* ^132/135^La^3+^ biodistribution in ICR mice (n = 3, mean ± SD) immediately following the last timepoint PET imaging, measured by gamma counting.

*Ex vivo* biodistribution studies were carried out at 20 h p.i. to validate PET data and obtain a more detailed distribution profile of ^132/135^La^3+^. Corroborating the results of the PET imaging, a high accumulation of the radiometal in liver and bone (30.45 ± 2.62 %IA g^−1^ and 6.65 ± 0.42 %IA g^−1^; n = 3) was observed (Fig. 5). The results of both PET imaging and biodistribution studies show good agreement in trend and magnitude with previous reports showing a preferential *in vivo* distribution of La^3+^ ions to the liver and the skeleton^13^.

These findings confirmed the feasibility of using the positron-emitting ^132^La to track longitudinal *in vivo* biodistribution of free La^3+^ and La compounds noninvasively with PET imaging. Additionally, this work also suggests the use of ^132^La as a diagnostic congener of therapeutic radionuclides currently of intense interest, such as ^135^La and ^225^Ac, given the chemical similarities between these radiometals^14^.

## Conclusion

The methods described above achieve the highest chemical purity, apparent molar activity, and Ba/La separation factor yet reported for the production of ^135^La using ^nat^Ba targets. More importantly, to the best of our knowledge, this is the first study that employs the co-produced ^132^La to monitor the *in vivo* biodistribution of the therapeutic Auger-emitting ^135^La using PET imaging. Further studies exploring isotopically enriched targets for ^135^La-based targeted radionuclide therapy are warranted.

## Supplementary information

Supplementary Information
