# Supplementary material for: Production and in vivo PET/CT imaging of the theranostic pair 132/135La
Source: Sci Rep. 2019 Jul 23;9:10658. doi: 10.1038/s41598-019-47137-0 (PMC6650468; doi:10.1038/s41598-019-47137-0)
Supplement: Supplementary file 1 — Supplementary Information [file 41598_2019_47137_MOESM1_ESM.docx]

**Production and *in vivo* PET/CT imaging of the theranostic pair ^132/135^La**

Eduardo Aluicio-Sarduy^a,*^, Reinier Hernandez^b^, Aeli P. Olson^a^, Todd E. Barnhart^a^; Weibo Cai^b^, Paul A. Ellison^a^ and Jonathan W. Engle^a,b^

*^a^Department of Medical Physics, University of Wisconsin-Madison, Madison, WI 53705, USA*

*^b^Department of Radiology, University of Wisconsin-Madison, Madison, WI 53705, USA*

**Supplementary Information**


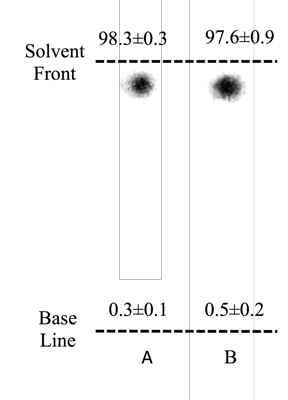


**Figure S1:** Radio-TLC analysis of (A) ^132/135^LaCl_3_ in 0.1 M HCl, (B) injected formulation (pH=6-6.5). Silica-impregnated paper was used as stationary phase and 50mM EDTA as the mobile phase. Under these conditions, the “free” ^132/135^La^3+^ ions move with the solvent front, while any formed radiocolloid remains at the baseline. In experiments not presented here, ^132/135^La^3+^ formulations at higher pH (>8.0) produce spots with R_f_ = 0 using the same rTLC method. Values are reported as mean ± SD (n=3)

**Table S1:** Production yields of ^132/133/135^La at different proton irradiation energies (n=3). Reported values are EOB corrected

| **Beam energy (MeV)** | **Production yields (MBq/µAh)** | | |
| --- | --- | --- | --- |
|  | **^132^La** | **^135^La** | **^133^La** |
| **11.9** | 0.26 ± 0.05 | 5.6 ± 1.1 | < 0.08 |
| **16** | 0.48 ± 0.06 | 16.4 ± 1.1 | 6.5 ± 0.2 |

**Table S2:** ^132/135^La^3+^ tissue uptake quantification of hand-drawn PET VOIs in ICR mice (n = 3, mean ± SD) injected with a rapid intravenous bolus of ^132/135^La^3+^

| **Tissue** | **Uptake (%IA/g)** | | | |
| --- | --- | --- | --- | --- |
|  | **0.5h p.i.** | **2.5h p.i.** | **5h p.i.** | **20h p.i.** |
| Blood/Heart | 3.10 ± 0.28 | 2.80 ± 0.14 | 2.75 ± 0.20 | 2.45 ± 0.07 |
| Muscle | 1.01 ± 0.24 | 1.01 ± 0.10 | 0.94 ± 0.08 | 0.73 ± 0.07 |
| Bone | 5.23 ± 0.33 | 5.08 ± 0.50 | 5.11 ± 0.49 | 5.12 ± 0.76 |
| Liver | 26.08 ± 1.95 | 29.28 ± 2.32 | 29.27 ± 1.91 | 29.09 ± 1.39 |
| Kidney | 2.70 ± 0.46 | 1.93 ± 0.50 | 1.80 ± 0.30 | 1.74 ± 0.55 |
